# Role of B lymphocyte ratio in development of type 2 diabetes mellitus: results of a 7-year follow-up study

**DOI:** 10.3389/fendo.2025.1559052

**Published:** 2025-05-26

**Authors:** Dan-Ting Shen, Zhong-Hong Qie, Lan-Jing Zhao, Li-Juan Pan, Su-Dan Wang, Chun-Xing Liu

**Affiliations:** ^1^ Department of Laboratory medicine, Shanghai Health and Medical Center, Huadong Sanatorium, Wuxi, Jiangsu, China; ^2^ Department of Ophthalmology and Otolaryngology, The Affiliated Wuxi People’s Hospital of Nanjing Medical University, Wuxi People’s Hospital, Wuxi, Jiangsu, China; ^3^ Nanchang Center for Disease Control and Prevention, Nanchang, China

**Keywords:** B lymphocyte ratio, type 2 diabetes mellitus, cohort study, the mediation effect, B cell

## Abstract

**Objective:**

To investigate the association between B lymphocyte ratio (BLR) and type 2 diabetes mellitus (T2DM) outcome among healthy people.

**Methods:**

A retrospective study cohort was constructed based on healthy people who participated in annual physical examination in Shanghai Health and Medical Center from 2013 to 2020. For each patient, we collected data at the first physical examination in 2013. The Cox proportional risk regression model was used to analyze the association between BLR and the risk of T2DM. The mediating effect of traditional metabolic factors were further explored.

**Results:**

The study included 1505 participants with a mean age of 48.77 ± 8.33 years at baseline and a follow-up duration of 7.36 ± 0.99 years. During follow-up, a total of 72 new T2DM cases were identified (7.9/1000 person-years). After adjusted for confounders, the results showed that the participants with higher level of BLR (Quartile 4) had a doubling of the risk of T2DM when compared to those with lower BLR level (Quartile 1). The association of BLR with the risk of T2DM remained robust when patients with hypertension or patients with obesity were excluded. In addition, traditional metabolic factors including HDL-C and LDL-C partially mediated the association between BLR and the risk of T2DM.

**Conclusion:**

Elevated BLR level is significantly associated with a higher risk of T2DM development. HDL-C and LDL-C partially mediated the association between BLR and T2DM risk. Our research may have the potential to provide new therapeutic targets for the treatment of T2DM.

## Introduction

Diabetes mellitus (DM) has become a major global public health concern. As of 2021, an estimated 529 million people of all ages were living with diabetes (1). Type 2 diabetes mellitus (T2DM) represents nearly 90% of diabetes mellitus (2, 3). In 2020, a large-scale population-based survey indicated that the overall prevalence of diabetes in mainland China was 11.2%, which means China had the largest number of adults (around 112.5 million) with T2DM in the world (4).

Components of the immune system are altered in type 2 diabetes mellitus, with changes occurring in circulating leukocytes (5). Immunological changes include altered levels of specific cytokines, changes in the number and activation state of various leukocyte populations (5), which suggest that chronic inflammation is a potential mechanism underlying type 2 diabetes mellitus (6–8).

B cells have a considerable effect on the inflammatory environment. They are one of the crucial lymphocyte populations (9) and play roles in both innate and adaptive immunity through antibody production, cytokine secretion and interaction with T cells (both activating and inhibitory roles) (10). Accumulating evidence demonstrated that B lymphocytes promote insulin resistance by presenting antigens to T cells and producing pathogenic IgG antibodies (11). Previous study has revealed that B cell potentially contributes to overall higher inflammation in obese diabetic subjects (12), however, whether or not B lymphocyte ratio (BLR) levels influence the development of T2DM among healthy people remains unclear. Therefore, we performed this study to examine the association of B lymphocyte ratio with the risk of type 2 diabetes mellitus among healthy people in China.

## Methods

### Participants

**Figure 1:**
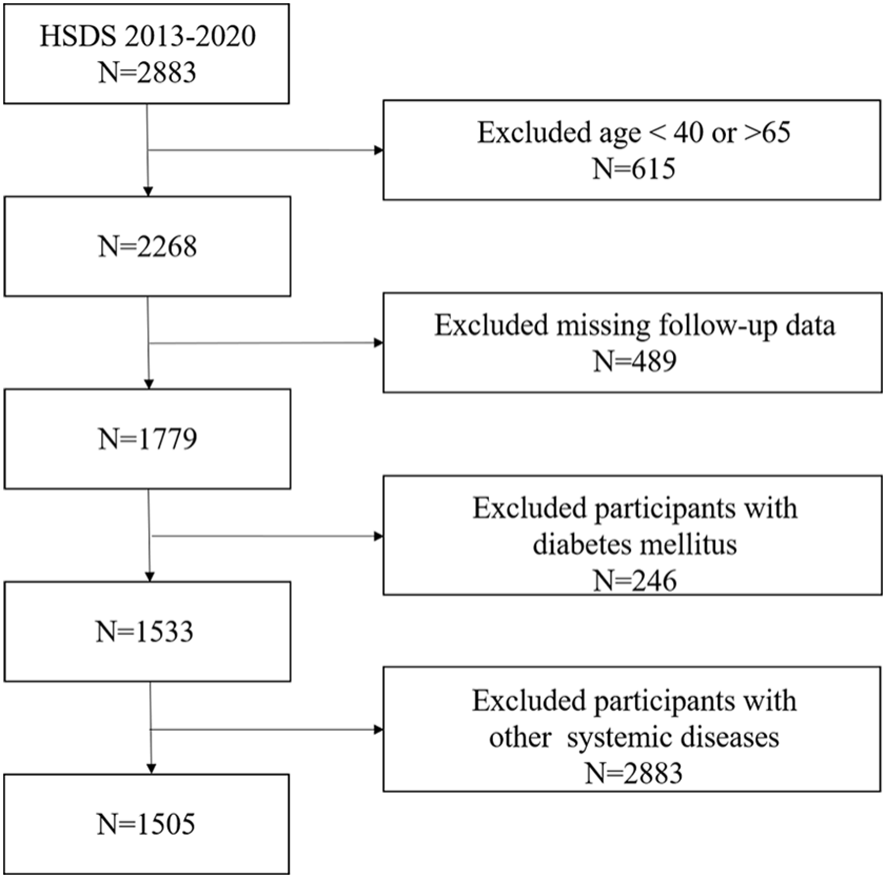
Flowchart of study selection.

Study participants were all from Huadong Sanatorium Diabetes Study Cohort (HSDS). HSDS is a retrospective study cohort based on healthy people who participated in annual physical examination in Shanghai Health and Medical Center from 2013 to 2020. In this study, a total of 2883 participants were enrolled and all of them completed the baseline examination in 2013. Follow-up surveys, including physical examinations, laboratory measurements and occurrence of outcomes were carried out annually. The inclusion criteria of this study were: (1) People aged 40 to 65 years; (2) People who had eight years of consecutive participation of physical examination; (3) People who had BLR data;(4) People who had no history of diabetes mellitus in the first physical examination. In the cohort, we excluded participants who had a history of cardiovascular disease, severe liver or kidney disease, chronic respiratory disease, autoimmune disease or tumor before enrolment; were missing follow-up data; or did not have data for mediators at baseline. Finally, 1505 people in total were participated in our research (**Figure 1**).

### Measurement of variables

Human samples were obtained after written informed consent under a protocol approved by Shanghai Health and Medical Center and conducted in accordance with the Declaration of Helsinki. We analyzed cells from people included in our study. Fasting venous blood samples were collected, and red blood cell count (RBC), white blood cell count (WBC), and neutrophil count (NEUT) were detected using a blood analyzer (Sysmex). The BD FACSCanto II flow cytometer was used to detect B lymphocyte ratio (BLR), T lymphocyte ratio (TLR), natural killer cell ratio (NKR), helper T lymphocyte ratio (THR) and suppressor T lymphocyte ratio (TSR). Biochemical tests including serum total cholesterol (TC), triglycerides (TG), high-density lipoprotein cholesterol (HDL-C), low-density lipoprotein cholesterol (LDL-C), fasting plasma glucose (FPG) and C-reactive protein (CRP) were measured on a AU5800 analyzer (Beckman Coulter) with commercial reagents.

### Data extraction

For each patient, we obtained data on age, sex, body-mass index (BMI), blood pressure, alcohol consumption, smoking status and medical history from their baseline examinations. Blood pressure was measured three times with a 5-minute interval between each measurement, and the average of the three measurements was calculated. All participants included in the study underwent annual follow-up health examinations from 2013 to 2020, with the diagnosis of type 2 diabetes mellitus as the outcome event.

### Definitions

Diabetes Mellitus patients were defined as: (1) Occasional plasma glucose value of ≥ 200 mg/dl (≥ 11.1 mmol/l); (2) Fasting plasma glucose of ≥ 126 mg/dl (7.0 mmol/l) (fasting time 8–12 h); (3) OGTT 2-h value in venous plasma ≥ 200 mg/dl (≥11.1 mmol/l); (4) HbA1c ≥ 6.5% (≥48 mmol/mol Hb) (5); On one or more diabetes medications (13). Smoking status was defined as an average daily consumption of more than one cigarette and continuous smoking for at least one year. Alcohol consumption was defined as drinking at least once a week on average and continuous drinking for no less than one year. Hypertension was defined as a systolic blood pressure (SBP) ≥ 140 mmHg and/or a diastolic blood pressure (DBP) ≥ 90 mmHg. Elevated levels of total cholesterol (TC), low-density lipoprotein cholesterol (LDL-C), and triglycerides (TG) were defined as ≥ 5.2 mmol/L (200 mg/dL), ≥ 3.4 mmol/L (130 mg/dL), and ≥ 1.7 mmol/L (150 mg/dL), respectively. A reduced level of high-density lipoprotein cholesterol (HDL-C) was defined as < 1.0 mmol/L (40 mg/dL).

### Statistical analysis

In this study, participants were divided into four groups according to the quartiles of BLR values. Quartile 1 (Q1) with BLR values below 9.60%. Quartile 2 (Q2) with BLR values between 9.60% and 12.06%. Quartile 3 (Q3) with BLR values between 12.07% and 14.95%. Quartile 4 (Q4) with BLR values above 14.95%. While categorical variables were shown as percentages, continuous variables were summarized using the mean and standard deviation. The differences between BLR (quartiles) were analyzed using weighted t-test or Wisxon test for continuous variables, and the chi-square for categorical variables. The log-rank test was used to analyze the differences in the cumulative incidence rate of T2DM among different BLR level groups. Three Cox proportional hazards regression models with progressive adjustment for covariates to estimate hazard ratios (HRs) and 95% confidence intervals (CIs) were used for T2DM events associated with quartiles of BLR. Model 1 adjusted for age and sex. Model 2 additionally adjusted for smoking status and alcohol consumption on the basis of Model 1. Model 3 included adjustment for the variables in Model 2 and BMI, TC, FPG, CRP and hypertension. To assess whether the inclusion of individuals with hypertension or obesity would affect model estimates, sensitivity analyses were performed. Mediation models were developed to investigate the potential mediation effects of serum TC, TG, LDL-C, HDL-C, FPG and body mass index (BMI) on the association between BLR level and T2DM risk. Mediation analyses were performed using R package bruceR. Total, direct and indirect effect estimates were calculated using multivariate adjusted linear regression analysis (adjusted for the potential confounding variables as described above). Analyses were performed using R version 4.0.5 and Statistical Analysis Software (SAS). All reported p values were two-sided and were deemed significant if less than 0.05.

### Role of the funding source

The funder of the study had no role in study design, data collection, data analysis, data interpretation, or writing of the report. The corresponding author had full access to all the data in the study and had final responsibility for the decision to submit for publication.

## Results

### Study population

The study included 1505 participants, of which 1058 (70.3%) were male, with a mean age of 48.77 ± 8.33 years at baseline and a follow-up duration of 7.36 ± 0.99 years. Participants with higher BLR levels were more likely to be males and smokers. Moreover, participants with higher BLR levels tended to have higher concentrations of WBC, NEUT and THR but lower concentrations of HDL-C, NKR, TLR and TSR (**Table 1**).

**Table 1:** Baseline characteristics among participants grouped according to the BLR quartile.

Baseline characteristics	BLR quartile				F/X^2^	P
	Q1 (N = 376)	Q2 (N = 377)	Q3 (N = 376)	Q4 (N = 376)		
Age at start of study (years)						
Mean (SD)	48.66 (8.43)	49.00 (8.29)	48.13 (8.35)	49.30 (8.21)	1.37	0.250
Sex						
Male	276 (73.4%)	267 (70.8%)	243 (64.6%)	272 (72.3%)	8.33	0.040
BMI (kg/m^2^)						
Mean (SD)	24.28 (2.83)	24.38 (2.55)	24.55 (2.65)	24.67 (2.58)	1.63	0.180
Smoking behavior						
	125 (33.2%)	140 (37.1%)	161 (42.8%)	201 (53.5%)	35.87	<0.001
Drinking status						
	181 (48.1%)	181 (48.0%)	167 (44.4%)	165 (43.9%)	2.36	0.502
SBP (mmHg)						
Mean (SD)	122.06 (15.11)	121.66 (14.55)	119.91 (15.01)	120.56 (14.09)	1.7	0.164
DBP (mmHg)						
Mean (SD)	75.11 (9.44)	74.86 (10.08)	73.90 (9.51)	74.4 (9.76)	1.14	0.332
Hypertension						
	112 (29.8%)	103 (27.3%)	114 (30.3%)	106 (28.2%)	1.06	0.786
FPG (mmol/L)						
Mean (SD)	5.43 (0.48)	5.45 (0.46)	5.38 (0.47)	5.41 (0.47)	1.57	0.196
TG (mmol/L)						
Mean (SD)	1.55 (1.57)	1.57 (1.01)	1.66 (1.27)	1.61 (0.89)	0.61	0.607
TC (mmol/L)						
Mean (SD)	5.07 (1.01)	5.12 (0.87)	5.14 (0.92)	5.09 (0.86)	0.46	0.708
LDL-C (mmol/L)						
Mean (SD)	3.05 (0.82)	3.14 (0.76)	3.17 (0.79)	3.20 (0.77)	2.53	0.056
HDL-C (mmol/L)						
Mean (SD)	1.31 (0.34)	1.29 (0.33)	1.24 (0.32)	1.20 (0.32)	8.02	<0.001
RBC (10^12^/L)						
Mean (SD)	4.91 (0.39)	4.91 (0.42)	4.87 (0.43)	4.91 (0.45)	0.71	0.545
WBC (10^9^/L)						
Mean (SD)	5.99 (1.34)	6.09 (1.41)	6.23 (1.64)	6.60 (1.82)	11.16	<0.001
NEUT (10^9^/L)						
Mean (SD)	3.30 (1.06)	3.36 (1.07)	3.48 (1.23)	3.66 (1.31)	7.02	<0.001
BLR (%)						
Mean (SD)	7.57 (1.58)	10.79 (0.75)	13.46 (0.85)	18.37 (2.98)	2502.54	<0.001
TLR (%)						
Mean (SD)	69.23 (10.28)	68.67 (9.05)	67.95 (8.10)	65.24 (7.58)	15.16	<0.001
THR (%)						
Mean (SD)	37.57 (8.75)	38.75 (8.07)	39.5 (7.68)	38.85 (7.13)	4.00	0.008
TSR (%)						
Mean (SD)	28.91 (9.18)	27.49 (7.94)	26.13 (6.82)	24.48 (6.80)	22.47	<0.001
NKR (%)						
Mean (SD)	22.43 (10.28)	19.93 (8.93)	17.96 (8.24)	15.7 (7.24)	40.59	<0.001
CRP (mg/L)						
Mean (SD)	1.72 (4.46)	1.46 (3.42)	1.45 (2.38)	1.66 (3.08)	0.7	0.55

THR, helper T lymphocyte ratio; TSR, suppressor T lymphocyte ratio.

Q1: BLR <9.60%;

Q2: BLR 9.60%-12.06%;

Q3: BLR 12.07%-14.95%;

Q4: BLR >14.95%.

### Association between BLR and risk of T2DM

**Figure 2:**
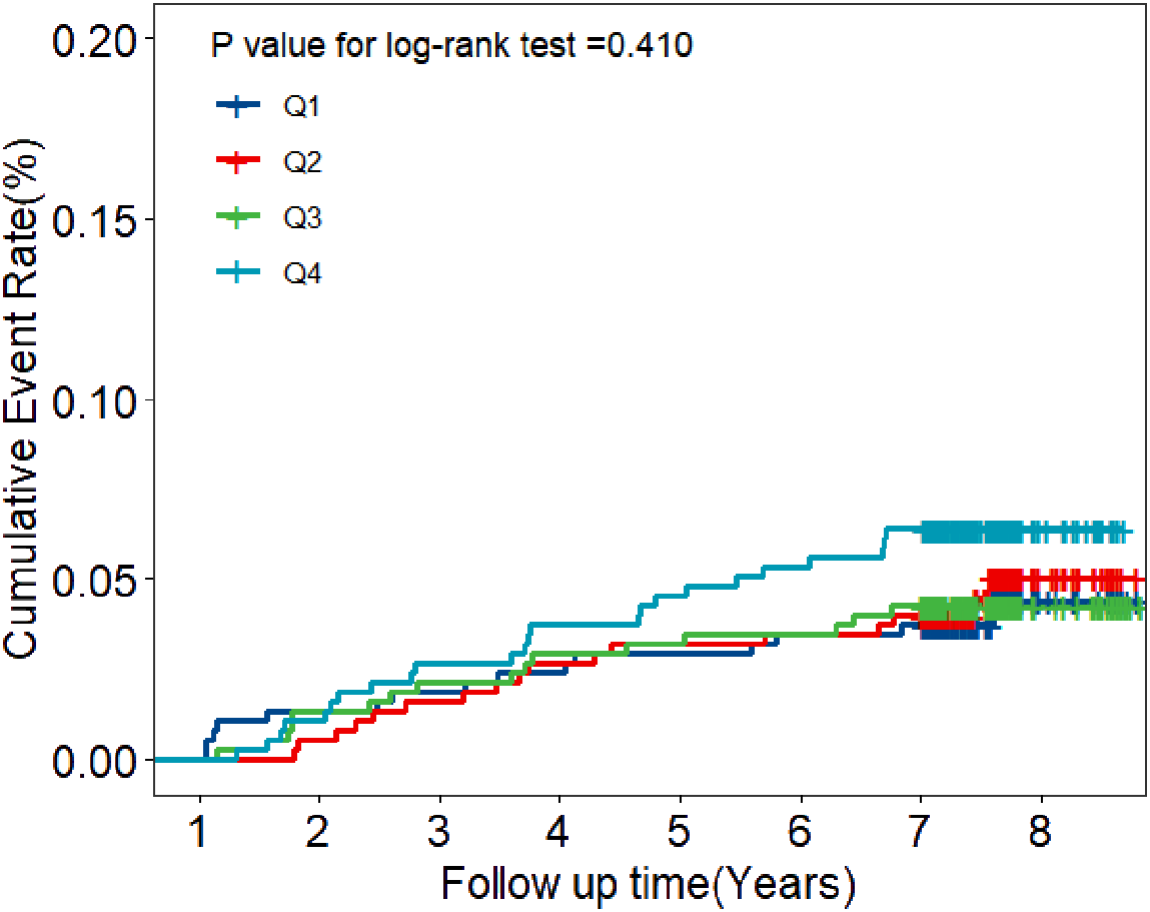
Kaplan-Meier estimates of cumulative incidence of T2DM according to the quartiles of BLR. Navy blue represents Quartile 1 with BLR values below 9.60; Red represents Quartile 2 with BLR values between 9.60 and 12.06; Green represents Quartile 3 with BLR values between 12.07 and 14.95; Light blue represents Quartile 4 with BLR values above 14.95.

**Figure 3:**
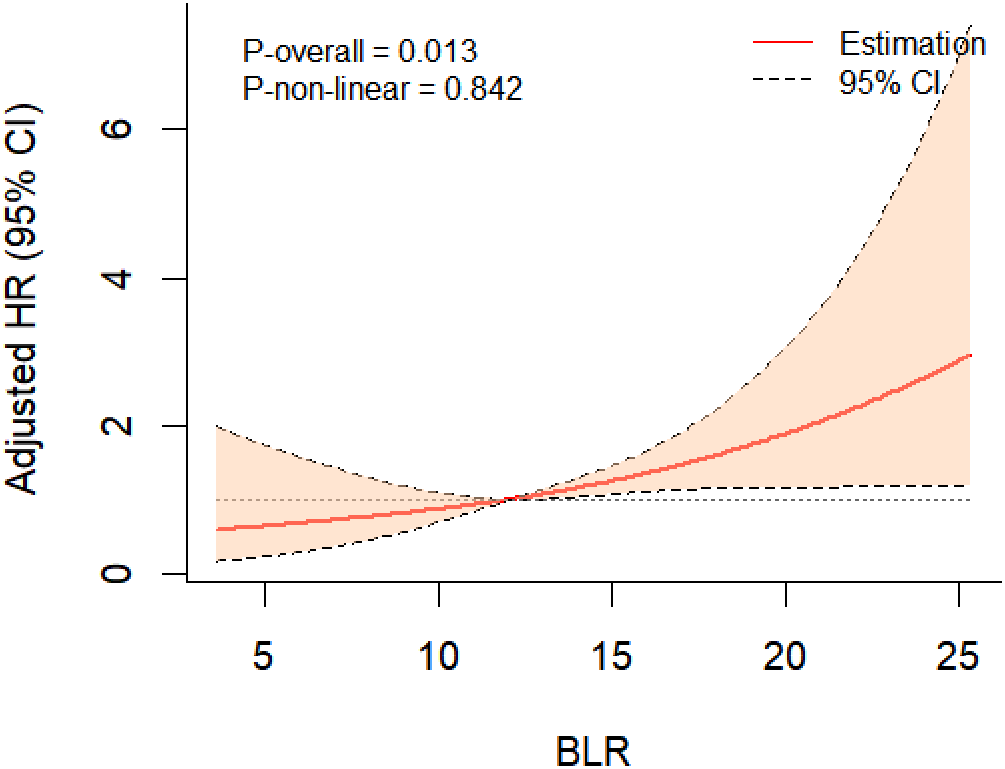
RCS analysis showing a liner dose-response association between BLR and T2DM risk.

During the eight years of follow-up, T2DM developed in 72 participants (7.9/1000 person-years). Kaplan-Meier plots showed that individuals with a BLR < 9.60% (the lowest quartile) had the lowest cumulative incidence of T2DM, however, those with a BLR > 14.95% (the highest quartile) had the highest cumulative incidence of T2DM (**Figure 2**). RCS analysis further implicated a liner dose-response association between BLR and T2DM risk (**Figure 3**). The risk of T2DM rose slightly with increasing BLR across the range from 5% to 25%.

In model adjusted only for baseline age and gender, the hazard ratio for T2DM per increase of 1 in the BLR was 1.06 (95% CI, 1.01 to 1.11) (**Table 2**, model 1). In comparison with subjects in Quartile 1, the hazard ratio for T2DM was 1.14 (95% CI, 0.57 to 2.29) in Quartile 2, 1.16 (95% CI, 0.57 to 2.35) in Quartile 3 and 1.62 (95% CI, 0.85 to 3.10) in Quartile 4 (**Table 2**, model 1). The hazard ratio for T2DM associated with BLR was nearly identical in model incorporating smoking status and alcohol consumption (**Table 2**, model 2) to that shown in model 1 in **Table 2**. After adjustment for baseline age, gender, BMI, smoking status, alcohol consumption, hypertension, FPG, CRP and TC, there was an increase in the risk of T2DM of 9 percent for each increment of 1 in BLR value (**Table 2**, model 3). As compared with Quartile 1, there was a 17 and 36 percent greater risk of T2DM in Quartile 2 and Quartile 3, respectively. Quartile 4 had a doubling of the risk of T2DM (**Table 2**, model 3).

**Table 2:** Associations between the BLR levels and T2DM risk and sensitivity analysis.

BLR quartile	N (Cases)	Model 1 HR (95% CI)	Model 2 HR (95% CI)	Model 3 HR (95% CI)	P
Q 1	376 (15)	1.00	1.00	1.00	1.000
Q 2	377 (17)	1.14 (0.57, 2.29)	1.17 (0.58, 2.34)	1.17 (0.57, 2.41)	0.676
Q 3	376 (16)	1.16 (0.57, 2.35)	1.27 (0.62, 2.59)	1.36 (0.66, 2.81)	0.405
Q 4	376 (24)	1.62 (0.85, 3.10)	1.88 (0.97, 3.65)	2.45 (1.21, 4.96)	0.013
P for trend^a^	0.159				0.012
per increment of 1		1.06 (1.01, 1.11)	1.07 (1.02, 1.12)	1.09 (1.03, 1.14)	
Excluding participants with hypertension					
Q 1	269 (8)	1.00	1.00	1.00	1.000
Q 2	267 (9)	1.16 (0.45, 3.01)	1.15 (0.44, 2.99)	1.80 (0.66, 4.90)	0.248
Q 3	268 (8)	1.10 (0.41, 2.93)	1.23 (0.45, 3.31)	1.51 (0.55, 4.16)	0.428
Q 4	266 (13)	1.56 (0.65, 3.77)	1.78 (0.73, 4.38)	2.49 (0.96, 6.51)	0.062
P for trend^a^	0.291				0.088
per increment of 1		1.05 (0.98, 1.12)	1.07 (1.00, 1.14)	1.08 (1.01, 1.16)	
Excluding participants with obesity					
Q 1	343 (12)	1.00	1.00	1.00	1.000
Q 2	344 (13)	1.10 (0.50, 2.42)	1.16 (0.53, 2.54)	1.49 (0.65, 3.39)	0.343
Q 3	343 (15)	1.36 (0.64,2.92)	1.53 (0.71, 3.31)	1.57 (0.72, 3.43)	0.257
Q 4	343 (19)	1.61 (0.78, 3.32)	1.96 (0.93, 4.12)	2.39 (1.08, 5.28)	0.031
P for trend^a^	0.170				0.035
per increment of 1		1.06 (1.01, 1.12)	1.08 (1.02, 1.14)	1.09 (1.03, 1.15)	

a Test for linear trend was performed using the median BLR levels for each quartile as a continuous variable.

BLR, B lymphocyte ratio; Q1, Q2, Q3, and Q4 were the quartiles of BLR.

Model 1 was adjusted for baseline age and gender.

Model 2 was adjusted for baseline age, gender, smoking status and alcohol consumption.

Model 3 was adjusted for baseline age, gender, BMI, smoking status, alcohol consumption, hypertension, FPG, CRP and TC.

### Sensitivity analysis

When excluding participants with hypertension, in model adjusted only for baseline age and gender, the hazard ratio for T2DM per increase of 1 in the BLR was 1.05 (95% CI, 0.98 to 1.12) (**Table 2**, model 1). In comparison with subjects in Quartile 1, the hazard ratio for T2DM was 1.16 (95% CI, 0.45 to 3.01) in Quartile 2, 1.10 (95% CI, 0.41 to 2.93) in Quartile 3 and 1.56 (95% CI, 0.65 to 3.77) in Quartile 4 (**Table 2**, model 1). The hazard ratio for T2DM associated with BLR was similar in model 2 to that shown in model 1. After adjustment for baseline age, gender, BMI, smoking status, alcohol consumption, hypertension, FPG, CRP and TC, there was an increase in the risk of T2DM of 8 percent for each increment of 1 in BLR value (**Table 2**, model 3). The risk of developing T2DM was 2.49 times higher in the Quartile 4 compared to the Quartile 1 (**Table 2**, model 3).

After excluding participants with obesity, in model adjusted only for baseline age and gender, the hazard ratio for T2DM per increase of 1 in the BLR was 1.06 (95% CI, 1.01 to 1.12) (**Table 2**, model 1). In comparison with subjects in Quartile 1, the hazard ratio for T2DM was 1.10 (95% CI, 0.50 to 2.42) in Quartile 2, 1.36 (95% CI, 0.64 to 2.92) in Quartile 3 and 1.61 (95% CI, 0.78 to 3.32) in Quartile 4 (**Table 2**, model 1). The hazard ratio for T2DM associated with BLR was slightly higher in model2 to that shown in model 1. In model 3, as compared with Quartile 1, there was a 49 and 57 percent greater risk of T2DM in Quartile 2 and Quartile 3, respectively. Quartile 4 still had the highest risk of T2DM among four groups (**Table 2**, model 3).

**Figure 4:**
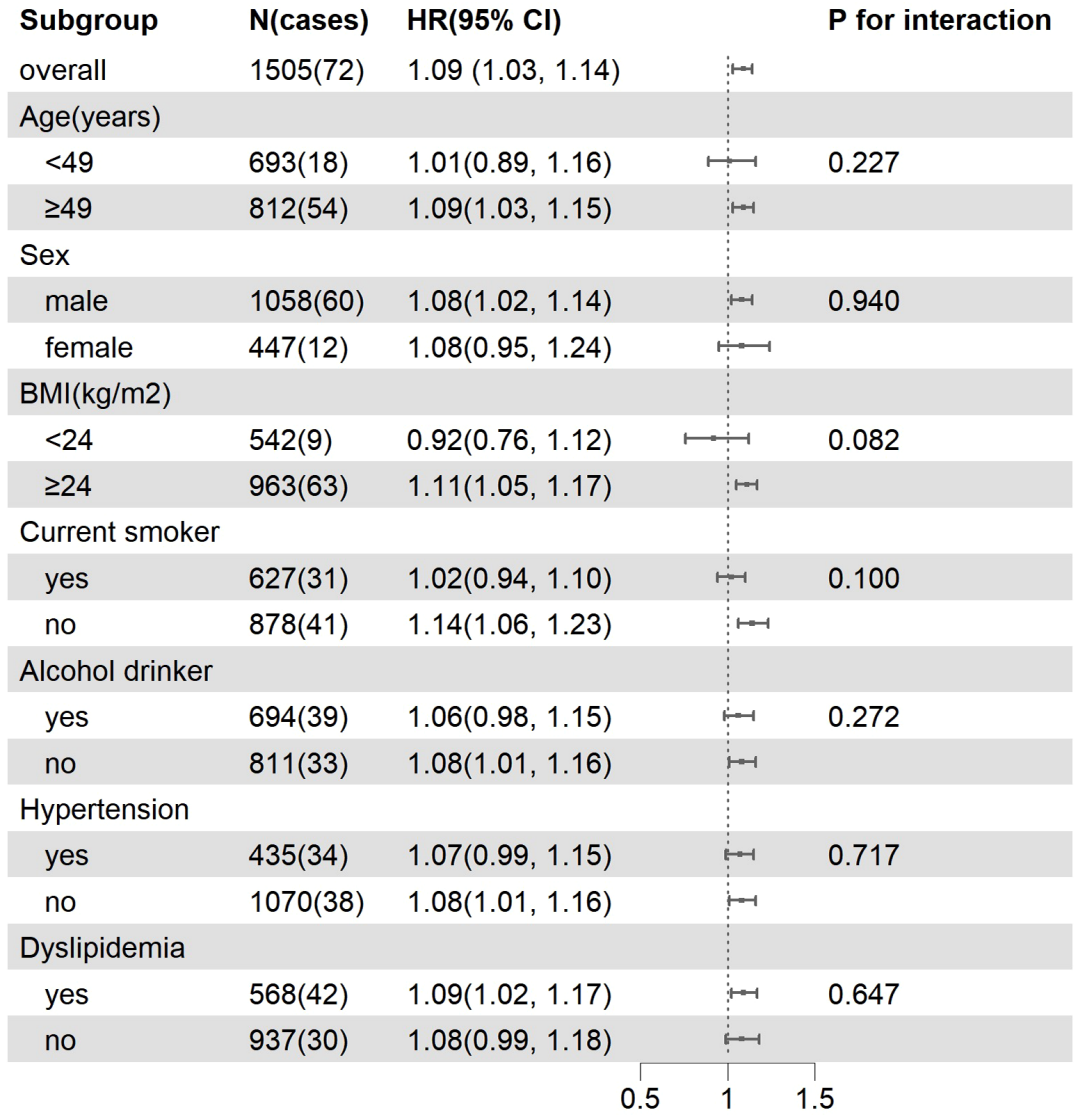
Subgroup analysis of the association between BLR and incident T2DM. Model were adjusted for baseline age, gender, BMI, smoking status, alcohol consumption, hypertension, FPG, CRP and TC.

Sensitivity analysis indicated that the association of BLR with the risk of T2DM remained robust when patients with hypertension or patients with obesity were excluded. In addition, the stratified analysis showed that the association between BLR and the risk of T2DM were not different in different subgroups (**Figure 4**).

### Mediating effect analysis

Results from the mediation analysis results were shown in **Table 3**. There was a 16.6% of positive mediation effect of LDL-C on the association between BLR and T2DM risk (indirect effect: 0.0041, 95% CI: 0.0006 to 0.01). However, a negative mediating effect of HDL-C on the association between BLR and T2DM risk was 15.4% (indirect effect: -0.0077, 95% CI: -0.0152 to 0.00).

**Table 3:** Assessment of mediating effects of BMI, TC, TG, HDL-C, LDL-C and FPG on the association between BLR levels and T2DM incidence.

Mediators^a^	Indirect effect (95% CI)	Direct effect (95% CI)	Total effect	Mediating effects (in %)
BMI	0.0042 (0.0006, 0.01) *	0.0689 (0.0170, 0.12) *	0.0730 (0.0217, 0.13) *	5.4
TG	0.0017 (-0.0032, 0.01)	0.0937 (0.0433, 0.14) *	0.0954 (0.0435, 0.15) *	1.4
TC	0.0009 (-0.0152, 0.00)	-0.0075 (-0.0557, 0.04)	-0.0065 (-0.0530, 0.04)	0.3
HDL-C	-0.0077 (-0.0152, 0.00) *	-0.0260 (-0.0786, 0.03)	-0.0337 (-0.0830, 0.02)	15.4
LDL-C	0.0041 (0.0006, 0.01) *	0.0115 (-0.0352, 0.05)	0.0156 (-0.0301, 0.06)	16.6
FPG	-0.0009 (-0.0052, 0.00)	0.3770 (0.3395, 0.43) *	0.3761 (0.3347, 0.43) *	0.2

All analyses are adjusted for baseline age, sex, BMI, smoking status, alcohol consumption, hypertension, FPG, CRP and TC.

a Use Z-scores to standardize all mediators.

* P<0.05.

## Discussion

We corroborate previous studies (11) which implicate the role B cells played in the development of insulin resistance and T2DM. Our data suggest a pathogenic role for high-level BLR in the promotion of T2DM. The risk of T2DM significantly increases in the high-level BLR group compared to the low-level BLR group.

B-cells can be divided into B-1 and B-2 cells (14). B-1 cells and Bregs inhibit inflammation through production of natural IgM and IL-10, and B-2 cells promote inflammation through both inflammatory cytokine production and IgG production, respectively (9). C. Deng and his colleagues also found that B-1b cells had a negative correlation with HbA1c, in addition, the correlation of TG and B-2 cells with HbA1c were contrary to B-1b cells (15).

B cells regulate multiple components of the immune system. Previous study has revealed that B cell potentially contributes to overall higher inflammation in obese diabetic subjects (12). It is said that B cells contribute to insulin resistance by presenting antigens to T cells, secreting inflammatory cytokines, and producing pathogenic antibodies (14). They may infiltrate white adipose tissue (WAT) very early in response to high-fat feeding and worsen glucose metabolism (16), and then accumulate within the adipose tissue (17). B cells promote inflammation in obesity through multiple mechanisms (18). Immune dysregulation in adipose tissue of obese subjects results in a chronic low-grade inflammation (19). Low-grade chronic inflammation is now recognized to be a fundamental facet of T2DM (20).

Rapid economic development has led to a change in lifestyle among the Chinese population. The rise in obesity, a sedentary lifestyle and energy-dense diets have made China the major area with T2DM epidemic (2, 4). Given the same body mass index, Asians tend to have a higher total body fat percentage and greater abdominal obesity (21). Abdominal obesity induces the development of type 2 diabetes and is often accompanied by dyslipidemia with high levels of TG and LDL, and low levels of HDL (22). Our research suggested that BMI, HDL-c and LDL-c partially mediated the association between BLR and the risk of T2DM, which aligns with the existing literature that systemic inflammation is related to diabetic dyslipidemia (23).

As our study showed that BLR was an independent risk factor for the development of T2DM, B cell might be the potential therapeutic targets for T2D. B cell manipulation may be a novel approach to the treatment of obesity-related insulin resistance and potentially to the prevention of T2D (14). Winer and colleagues demonstrated that the therapeutic depletion of B lymphocytes in animal model protected against impaired glucose metabolism and increased insulin sensitivity (17). Monoclonal antibody (mAb) therapy directed against the CD20 cell surface molecule of human B cells has shown varying degrees of efficacy in treating B-cell malignancies and some autoimmune diseases (24, 25). It is also proved to be useful in the treatment of abnormal glucose metabolism (11). Anti-inflammatory agents including salsalate and IL-1 blockers, moderate the progression of diabetes by increasing insulin levels and β-cell function (26, 27).

Other B cell and antibody-modulating agents, including intravenous immunoglobulin (IvIg), transmembrane activator and calcium modulator and cyclophilin ligand (TACI) fusion proteins, and antibodies or small-molecule inhibitors to CD19, CD22, CD79a and b, B lymphocyte stimulator (BLyS), spleen tyrosine kinase (Syk) and a proliferation-inducing ligand (APRIL), are all suggested for new possible uses in the management of T2DM (11).

Our estimates might not be representative as the people enrolled in our research are all Chinese. Previous study showed that Asians are more susceptible to insulin resistance and diabetes than Western counterparts by genetic mechanisms (28). Those of Asian ethnicity have an increased predisposition for type 2 diabetes (20). A recent study also showed that Asian individuals whose BMI or waist circumference are the same with white population tend to have higher percentage of visceral adiposity (15).

However, limitations are as follows. Firstly, we did not distinguish the impact of different types of B lymphocytes (such as B1 cells and B2 cells) on the risk of developing T2DM and the absolute value of B cells are unknown; Secondly, we did not perform an oral glucose tolerance test, which may underestimate the incidence of T2DM; Thirdly, we did not investigate inflammatory factors other than CRP, such as IL-6, IL-10, and TNF- α; Finally, as the included population are from a retrospective cohort, there may be selection bias in this study.

## Conclusion

Our study provides new insights into the relationship between B lymphocyte ratio and T2DM. These results establish the importance of B cells in the etiology of type 2 diabetes. Novel therapeutic interventions are needed to improve the treatment of T2DM. Exploring whether manipulating B lymphocyte populations could be a potential therapeutic avenue for T2DM would provide a clear direction for clinical trials.

## Data Availability

The original contributions presented in the study are included in the article/supplementary material. Further inquiries can be directed to the corresponding author/s.
